# Racial biases in healthcare: Examining the contributions of Point of Care tools and unintended practitioner bias to patient treatment and diagnosis

**DOI:** 10.1177/13634593211061215

**Published:** 2021-12-07

**Authors:** Sachil Singh

**Affiliations:** Queen’s University, Canada

**Keywords:** ethnicity and health, primary care, race, social inequalities in health, technology in healthcare

## Abstract

Sophisticated algorithms are used daily to search through hundreds of medical journals in order to package updated medical insights into commercial databases. Healthcare practitioners can access these searchable databases—called Point of Care (PoC) tools—as downloadable apps on their smartphones or tablets to comprehensively and efficiently inform patient diagnosis and treatment. Because racist biases are unintentionally incorporated into the search reports that the companies generate and that practitioners regularly access, the aim of this article is to examine how healthcare practitioners’ “pre-existing” racial stereotypes interact with pithy conclusions about race and ethnicity in PoC tools. I use qualitative research methods (content analysis, discourse analysis, open-ended semi-structured interviews, and role play) to frame the analysis within the Public Health Critical Race Praxis (PHCRP). This approach facilitates an understanding of how biological racism—the use of scientific evidence to support inherent differences between races—that is embedded in PoC algorithms informs a practitioner’s assessment of a patient, and converges with persistent racial bias in medical training, medical research and healthcare. I contextualize the study with one semi-structured interview with an Editor of a leading PoC tool, MedScope (pseudonomized), and 10 semi-structured interviews with healthcare practitioners in S.E. Ontario, Canada. The article concludes that PoC tools and practitioners’ personal biases contribute to racial prejudices in healthcare provision. This warrants further research on racial bias in medical literature and curriculum design in medical school.

## Introduction

When I was a resident [in the 1990s], I was rotating with a school-based physician and we were in a high school that was mostly Latino and a girl came in with a sore throat. So I started taking her history and asking “have you been exposed to anybody sick? Does your throat hurt when you do this or that? Do you have a temperature?” And at some point I said to her “have you had oral sex?” And afterwards, the [attending] physician said to me “did you ask her that question because she was Latina?” And I had to admit to myself that I wouldn’t have necessarily asked that to a “White” teenager.

(Dr. Williams, MedScope PoC tool Editor, personal interview, 2018)

One problem that this excerpt captures is that when a healthcare practitioner’s “pre-existing” racial stereotypes inform patient care, diagnosis, and treatment can border on a genomic approach to racial and ethnic identity. Another related problem is that the significantly automated medical databases that practitioners draw on during patient care often contain misrepresented race data passed on from one source to the next in a way that resembles the children’s game “broken telephone” (Singh and Steeves, 2020). Consequently, racial biases can emerge in patient care that negatively affect patients. A further related problem is that the medical scholarship on race and ethnicity which populates these databases is sometimes conducted contrary to published guidelines (Small, 2014). What is less explored in the literature is how these problems—a practitioners’ pre-existing racial stereotypes and the race-related conclusions about medical conditions in significantly automated databases—relate to each other. Do practitioners inform themselves of how the medical studies that they rely on are performed? Are the research conclusions accepted at face value because they are accessed through reputable digital applications? How do healthcare practitioners interpret and act upon descriptions of race and ethnicity differently when patient diagnosis and treatment are informed *with and without* automated medical databases? Together, these questions inform the aim of this article, which is to examine how healthcare practitioners’ pre-existing racial stereotypes interact with pithy conclusions about race and ethnicity in Point of Care (PoC) tools in shaping patient treatment and diagnosis.

PoC tools are databases that are significantly automated providing healthcare practitioners and medical researchers with access to consolidated and searchable medical insights from hundreds of guideline organizations and medical journals. The stated goal of leading PoC tools, such as UpToDate (2021) and DynaMed (2021), is that they improve clinical decision-making by providing practitioners with “on-the-go” retrieval of medical literature (such as through an application on a smartphone) in an accessible and searchable format that can be used to aid patient treatment and diagnosis. Algorithms are key to the functioning of PoC tools because they are used for the daily automated searches of the literature for revisions or new insights on the thousands of topics contained in each PoC tool. Algorithms also facilitate a practitioner’s efficient searches for results using queries pertaining to specific medical conditions, drug doses, race, and ethnicity, etc. Therefore, PoC tools are increasingly ubiquitous in patient care because efficiency is key, especially in a practitioner’s consultation with a patient when the ability to access such information can inform accuracy of diagnosis and treatment as well as the number of patients cared for per day.

But when one takes a critical look at the conclusions about race and ethnicity that are presented in PoC tools like DynaMed, the focus shifts from the benefits of efficiency to the tool’s unintended promotion of racism. For example, the DynaMed entries that practitioners access show that: high susceptibility to sexually transmitted diseases like Chlamydia trachomatis is based on factors such as receptive anal sex (*social* behavior) and Latino ethnicity (*identity*). Similarly, insomnia in adults is associated with low socioeconomic status (*social condition*) and African American race (*identity*). Preterm labor and premature birth are most common in those who use tobacco (*social* behavior), have undergone a cervical excision (*medical procedure*), or are of Black race or Hispanic ethnicity (*identities*).^1^ As a final example, Vitamin D deficiency in children is associated with television, computer, or video use for more than 4 hours per day (*social* behavior) and non-Hispanic Black or Mexican American ethnicity (*identity*) (DynaMed entries).^2^ In all these cases, a focus on the intersection between social behavior/social condition and racial identity brings into question the legitimacy of biological race, or the use of scientific evidence to support “inherent differences between races.” Because the role of PoC tools in prejudicial patient care is underexplored in the literature, this article contributes to a broader literature on the deepening of stereotypes constructed on biological interpretations of the association between social behavior and social identity.

More specifically, this article contributes to such scholarship by focusing on what happens when the codification of racist conclusions in PoC tools converges with practitioners’ pre-existing prejudices. Building on Singh and Steeves (2020), I further argue that at the intersection of “new” technologies and “old” medical practice and beliefs is a reproduction of racial disparities and historical disadvantage. I do this by demonstrating that PoC tools, with the example of the MedScope PoC tool (pseudonomized), contribute to the layering of subjectivities that are inserted into health research. Additionally, healthcare practitioners have their own inconsistent understandings of race and ethnicity that not only point to the socially contingent meanings of the terms, but also highlight the dangerous ways in which algorithmically mobilized racist insights can be used to justify practitioners’ own prejudices.

## Situating the study in the literature

In medical practice, there is a growing interest in diagnostic algorithms that help to individualize patient risk assessment, such as by race. More generally, this can be contextualized in light of: the historical formation of racial knowledge which also had implications for the racialization of disease (Muhammad, 2011); and how racial hierarchies are amplified when race is encoded in automated software yet packaged to appear objective (e.g. Benjamin, 2019; Noble, 2018). To this end, correlational data in PoC tools often associate race and ethnicity with social behavior, social conditions, and physiological symptoms. While this might serve the interest of early detection of medical conditions, it can quickly stimulate racial profiling as seen when racialized patients disproportionately pay higher health insurance premiums, or experience higher general levels of social stigmatization, guilt, anxiety, and psychosocial stress compared to non-racialized patients (Foster et al., 2001: 235; Petersen, 2006: 488). In fact, research shows that the association of risks with group membership in already marginalized social categories cumulatively adds to risk statuses and likely experiences (e.g. Berthier-Foglar et al., 2012; Gandy, 2009; Garbarski, 2015; Seabrook and Avison, 2012; Veenstra and Patterson, 2015).

Despite these social risks, one of the key arguments in favor of including race and ethnicity in patient care is that human DNA correlates with already existing categories (Risch et al., 2002). This leads to the growing relationship between algorithms and previously siloed datasets to find undetected correlations. Building on this, some studies support the use of algorithms to predict ethnicity because of the view that this serves health equity. For example, Dalton et al. (2014) argue that cardiovascular disease (CVD) risk scores are helpful for predicting risk across ethnicities and are needed to serve the interests of health equity. Then there are those, like Elliott et al. (2008), who maintain that *self-reported* race/ethnicity is essential, but that the absence of this information can be substituted with data about surname and residential address. Contrary to both streams is a third, offered by Rosella et al. (2012), who remind readers that detailed ethnic information *does not* necessarily improve the accuracy of risk prediction tools for diabetes. Many race-adjusted algorithms legitimize race-based medicine by “guid[ing] decisions in ways that may direct more attention or resources to white patients than to members of racial and ethnic minorities” (Vyas et al., 2020: 874).

For example, the American Heart Association Get with the Guidelines–Heart Failure Risk Score categorizes Black patients as being at lower risk of death than “Nonblack” patients (p. 874). The estimated glomerular filtration rate (eGFR) predicts patients’ kidney function with higher eGRF values (for better kidney function) assigned to Black patients because they are “more muscular” (2020: 875). The Vaginal Birth after Caesarean (VBAC) algorithm predicts that African American and Hispanic women are more likely to be unsuccessful in a trial of labor if they previously underwent a Caesarean section. The STONE score predicts that Black patients are less likely to experience flank pain related to kidney stones (2020: 879). In all these cases, race is a key contributing factor in the personalized physical assessment of Black patients who are otherwise excluded from thorough evaluation of their conditions even before they might enter a consultation room (2020: 874–875, 879). As a result, these algorithms can perpetuate already existing racial inequalities.

Despite the expressed benefits of algorithmically driven “smart” technologies like PoC tools, some critics draw attention to the “black boxed” nature of proprietary algorithms (Pasquale, 2015) that often unintentionally contribute to social discrimination in unintended ways (Benjamin, 2019; Char et al., 2018; O’Neil, 2016). More particularly, the connection between smart technologies and historical racial and ethnic inequalities is a documented social problem where contributions already show how algorithms can deepen racial and ethnic oppression in intersecting areas of insurance (Gandy, 2010), policing (Ferguson, 2017), child welfare (Eubanks, 2018: 79, 127–173), and banking and credit scoring (Singh and Steeves, 2020). In all these areas, the role of human decision-making reduces such that inequality is increasingly automated.

In some cases, the algorithms are employed to predict ethnicity based on surnames. When surname algorithms in healthcare predict ethnicity based on the assumed majority race or ethnicity in a neighborhood, group trends are applied to individuals (Elliott et al., 2008: 1724). But group predictions are not always accurate, and are often discriminatory, at the individual level (e.g. Chow-White and Green, 2013; Iliffe and Manthorpe, 2004: 286; Gandy, 2009: 35–54; Ortega and Meyers, 2014: 17). When social discriminations such as those referred to above (insurance, policing, finance, surnames) are combined with those in healthcare, “cumulative disadvantage” characterizes the snowballing of social disadvantages for the same groups of people (Gandy, 2009).

Therefore, in the context of PoC tools that are designed to make race and ethnicity searchable, their algorithmic inclusion may create and deepen historical associations between health conditions and race and ethnicity. With an increasing algorithmic visibility of populations (Introna and Nissenbaum, 2000) comes the growing inclusion of social variables in commercial algorithms, often with systemic forms of inequality, such as race (McIlwain, 2017). Matamoros-Fernández (2017: 931) refers to this as “platformed racism,” which, in part, is the product of the “algorithmic shaping of sociability” (see also Noble, 2018). Because of the contributions of algorithms in these ways, scholars have documented key concerns with the racist outcomes of automated technologies (e.g. Benjamin, 2019; Eubanks, 2018; Noble, 2018). This article adds to such contributions with an examination of the underexplored roles of PoC tools and practitioner prejudices in racist patient outcomes.

## Method

This study employs qualitative research methods to address the aim of the research presented in the Introduction. Open-ended semi-structured interviews were conducted because they provided the necessary flexibility (open-ended) based on participants’ different medical specializations, while maintaining sufficient consistency (semi-structured) for comparison in the analysis. Content analysis and discourse analysis were employed throughout the interview process to detect recurring themes, connections between practitioners’ race-related prejudices and interpretations of PoC entries, to establish when saturation was reached in the study, and to problematize practitioners’ different uses of terminology. Role playing was also employed to simulate a complex racial context that challenges conceptions of biological race. This method was also used to compare how practitioners would deal with a presented circumstance differently in the face of the contested meanings of race and ethnicity in PoC tools.

This study builds on a previous one that performed a content analysis of the DynaMed PoC tool to understand its presentation of “race” and “ethnicity.” For this article, I conducted an open-ended, semi-structured interview with Dr. Williams (pseudonomized): a healthcare practitioner and MedScope (pseudonomized) PoC tool Editor of “systematic literature surveillance,” to understand how race and ethnicity are designed into PoC tools. I invited Editors of three PoC tools in total but only one responded positively. The purpose of interviewing PoC tool Editors was to gain a better understanding of the PoC platform design, and critical insights about the inclusion of race and ethnicity in data entries. Additionally, I conducted interviews with 10 healthcare practitioners (pseudonomized) in S.E. Ontario to assess how they interpret and act upon descriptions of race and ethnicity in PoC tools for patient treatment and diagnosis, as well as how PoC tool insights interact with their own pre-existing patient stereotypes. While there was overlap in the questions designed for practitioners and Editors pertaining to the operationalization of race and ethnicity, only practitioners were asked about their preferred PoC tools, how and why they use the tools, and their understandings of how the tools are designed. Questions for Editors were based on the design of their companies’ PoC tools, the balance in design between automation and human decision-making, and the extent to which the inclusion of race and ethnicity in PoC tools might (re)produce racist presentations of the medical literature.

To commence, I obtained ethical clearance from five research ethics boards from 2018 to 2019 (Children’s Hospital of Eastern Ontario, University of Ottawa, Queen’s University, Kingston Health Sciences Center, and the Georgian Bay Family Health Organization) in S. E. Ontario covering nine health institutions in the region. I recruited on the listservs of eight medical facilities (including hospitals and clinics) covered by these research ethics boards between July 2018 and March 2020. After a disappointing response rate in the first year (two participants), I amended my recruitment strategy to offer a $15 Starbucks gift card for participants’ time. This increased positive response rates three fold. Four healthcare practitioners canceled within 45 minutes of their respective scheduled interviews because of hospital emergencies in their wards. Unfortunately, they were unable to find additional free time for an interview. Nevertheless, the combination of reaching saturation on health practitioners’ uses of PoC tools with respect to race and ethnicity, together with the spread of the COVID-19 pandemic that increased the demands on healthcare workers, motivated the suspension of recruitment. Although the small sample size does not allow for the extrapolation of participants’ views to a broader population of practitioners, the in-depth interviews satisfied the qualitative exploration of the topic at hand.

Each interview was semi-structured, open-ended and lasted for up to 90 minutes. Participants were asked a series of questions to understand their pre-existing racial assumptions about their patients, determine the significance of PoC tools in their daily work, and to understand the amount of trust they place in such tools. Given how easily race and ethnicity are entangled in PoC tools (Singh and Steeves, 2020), participants were asked to unpack the meaning(s) attributed to these terms and to explain their medical relevance in their respective medical specializations.

In this study, I apply the Public Health Critical Race Praxis (PHCRP), which is a recent off-shoot of Critical Race Theory (CRT). Ford and Airhihenbuwa (2010) developed the PHCRP as a way to bridge CRT and public health theory. PHCRP allows the researcher to foreground social and health issues to promote social justice for marginalized populations. This approach has already been applied to a host of contexts ranging from police brutality (Gilbert and Ray, 2016), anti-racist medical school curriculum development (Hardeman et al., 2018), disparities research for health equity (Thomas et al., 2011), research practice in epidemiology (Allen and Lewis, 2020), and to understand the social construction of race with respect to Black women with endometrial cancer (Doll et al., 2018).

I adopt a race consciousness perspective, which is the backbone of the PHCRP (Ford and Airhihenbuwa, 2010: 1393), by situating myself in the study. This encourages critical self-reflection—a PHCRP trait—and helps to reveal one’s personal bias. Further, “[t]he reader is entitled to know something of the aims, expectations, hopes and attitudes that the writer brought to the field with him, for these will surely influence not only how he sees things but even what he sees” (Turnbull, 1972: 13). I positioned myself in this study because my personal experiences as a racialized South African born under apartheid are pointedly illustrative of an overarching argument in this article and is included in the context of advancing the literature. More specifically, as part of a broader effort to demonstrate the subjectivity of race, I changed my racial classification in South Africa to “White” in 2013, which presents me with an intentional dilemma that I brought to this study: when completing medical forms, or responding to a healthcare practitioner who asks for information about my race, do I label myself as Indian (apartheid classification applied generally to brown people), White (my “official” current classification in South Africa), or something else that conforms with the geopolitical context of the territory in which I find myself each time I am asked this question? This informed the role play in the interview schedule.

In this article, I apply the PHCRP according to its four main areas of focus:

(1) *Contemporary Patterns of Racial Relations* requires a description of societal racialization by time and space. These patterns are examined throughout my research process by considering the contextual factors in my conversations with participants.
(2) *Knowledge Production* requires an examination of the limits and/or biases of literature on algorithms and race. This is documented above in the Introduction.
(3) *Conceptualization and Measurement* calls for an explanation of how race is measured. This formed part of the interview schedule and the findings are reported in the next section.
(4) *Action* addresses what can be done to respond to racial inequities. Suggestions are offered in the Conclusion.

## Findings

### Operating a PoC algorithm

MedScope is a leading and widely used PoC tool that has been in operation for more than 20 years. In addition, MedScope prides itself on being “data based”—not data biased—because of the belief that it mobilizes the medical literature in an accessible, searchable and objective way (Personal Interview with a MedScope Editor, 2018). MedScope consists of at least 12 writers and editors (most of whom are non-clinical). Forty contracted specialists are employed to survey the most recent medical literature for updates to existing MedScope topics and to identify new studies to generate new medical topics. This process begins with an automated search of content of hundreds of medical journals and guideline organizations. This automation is enabled with a “proprietary blend of keywords” such as “randomized trial”, “systematic review,” filters to show every article with an abstract only etc. While the breadth of the data scoop is indicative of the powerful algorithms that fuel the daily data searches, in reality, the algorithms facilitate “use [of] whatever evidence there is even if it’s not the best and most unbiased” (Personal Interview with a MedScope Editor, 2018). Results from these searches are placed in an internal systematic literature management system from where each one is channeled to a contracted specialist (mostly made up of physicians) who examine each article to identify relevance and appropriateness for MedScope. This is a labor intensive process through which the contracted specialists generate feedback for topic writers regarding the topic to which the information belongs, the main points of the identified study, whether it reiterates content that already exists, or changes the basic tenets of how healthcare practitioners function. Writers take this information and create or amend a summary (including points about potential bias in the original study) and insert it into a topic. This summary is reviewed by a scientific editor before it is reviewed by a clinical editor, then copy-edited and published. This is the point at which healthcare practitioners, such as those introduced below, would access PoC tool data on devices like smartphones.

### Why do healthcare practitioners use PoC tools?

To better understand why healthcare practitioners use PoC tools to inform patient diagnosis and treatment, I asked them to justify their particular tool selections. Linked to this were questions about the relevance of PoC tools for patient diagnosis and treatment. Notably, none of the practitioners highlighted the functionality or design of their specific PoC tool over another as their motivation for selection. The main reason that practitioners use their selected PoC tool is that they have free access through their hospital affiliation, residency or as a university alumni perk. When these questions were broadened to ask about the perceived reliability of, and trust placed in, PoC tools in general, the responses were more varied. Dr. Zhao (Family Medicine) noted that “It’s an official app on my phone, so I believe it’s trustworthy. . .the hive mind of the profession says UpToDate is okay, so I trust it.” This “herd mentality” was shared by others. For example, Dr. Edwards (Family Medicine) trusts UpToDate “. . .simply because it’s peer-reviewed. . .I’m just willing to trust it. . .I can’t back up my statement any more than that,” while Dr. Hince (Pediatrics) also justified UpToDate: “I guess I would believe it would be quality information because of the mass of people that use it. If it wasn’t, someone would’ve spoke up. . .but I haven’t personally researched it.” Dr. Hince added, “We [physicians] trust PoC tools probably more than we should. . .[laughs]. . .but what else do we have?” Dr. Sampson (Family Medicine) was drawn by the subscription-based nature of Lexicomp, UpToDate and DynaMed: “The fact that it costs money makes me feel that someone is looking this stuff up and updating it. . ..” Dr. Jordan (Family Medicine) did not offer a justification for using her selected PoC tool except to state that “I just accept that it’s accurate.”

Another group of practitioners substantiated their views about the reliability of PoC tools in general by demonstrating a more in-depth overall understanding of the products. Dr. Bugwandeen (Emergency Medicine) has personally met a number of the contributors to PoC tools, so this removes some of the detachment that others might feel from using a well-known product to which they have no connection. Similarly, Dr. Freund (Allergy/Immunology) is familiar with a number of the experts who publish in PoC tools, but is always alert to questioning those publications. He does this by reading the primary literature on a given topic and comparing it to the content and publication date of PoC tool entries.

### Health practitioners’ approaches to race and ethnicity

In an effort to understand how healthcare practitioners interpret and act upon the meaning(s) attributed to race and ethnicity in PoC tools, I asked questions about the practical relevance of race and ethnicity to the respective fields of practice. I also asked about the value to the practitioners of specific conclusions in MedScope.

PoC tools present information about race and ethnicity inconsistently, reflecting concerns with both their publication practices and the framing of these terms in the broader medical literature (Singh and Steeves, 2020). It is therefore important to consider how healthcare practitioners define these terms and if their definitions compound the issue. It quickly became clear in the interviews that practitioners’ conceptual understandings of race and ethnicity varied drastically. On the one hand, their understandings of race ranged from geography and biology to skin color and genetics. Dr. Freund (Allergy/Immunology) believed that race is “not clinically relevant,” while Dr. Edwards (Family Medicine), who has a research background and is aware of how controversial this term is in medical research, opted “. . .to do a total cop-out” on this question. Despite the significance of the term to many branches of medical study, “race” is so definitionally obscure that one practitioner took 30 seconds to think about this question, while another took 50 seconds, before finally committing to responses.

On the other hand, perspectives on ethnicity were equally inconsistent. Dr. Hince (Pediatrics) believed that it is “a little bit of a mix of biologic [sic] and social construct, while Dr. Sampson (Family Medicine) viewed it as “class background, country of origin.” Again, some expressed uncertainty and did not have an answer. Others conflated race and ethnicity stating that they are both to do with genetics, and “By now calling it ‘race/ethnicity’ we are acknowledging that many of these are constructed.” Similarly, Dr. Roberts’ (Pediatrics) expressed that ethnicity is “. . .along the same lines” as race, and, “I don’t really know what to do with that. . .I prefer race, but even then. . .umm. . ..”

Given that healthcare practitioners operationalize race and ethnicity differently, their engagement with the terms in patient care is also expected to be different. This is explicated in the following section.

The terms “race” and “ethnicity” had the least relevance for Dr. Bugwandeen (Emergency Medicine) because “I deal with acute problems, not race and ethnicity.” He continued, “. . .you always have to be cognizant that you’re not trying to make a patient’s specific presentation fit into a box”. To the contrary, Dr. Zhao (Family Medicine) actively supported the collection and use of this data because they capture groups of “people with medical problems at a higher rate” that could therefore be treated pre-emptively. Dr. Hince (Pediatrics) questioned the relevance of race and ethnicity in healthcare, even when the motive is good:

It’s difficult these days to apply all those risk factors [of racialized and ethicized prevalence] because nobody is 100% from one racial background, everybody is very mixed, and so you can ask somebody what their race or ethnicity is and oftentimes they can’t really answer you or it’s like a big mix of lots of different backgrounds, and you’re [the doctor] not sure whether or not that really applies to this patient or it really doesn’t.

Dr. Hince’s view was shared by other practitioners, who reached the point of expressing knowledge of racial and ethnic susceptibility to various medical conditions. However, these practitioners added that they do not use this knowledge to influence their own approaches to patient treatment. But following a process of encouraging practitioners to self-reflect in the interviews, many had sudden realizations of instances where their actual practices differed from their initial recollection. For example, when explaining the relevance of race and ethnicity to her field, Dr. Freund (Allergy/Immunology) explained that “most of the time, I don’t really think it makes any difference” because “I completely base [my patient diagnoses and treatment plans] on symptoms.” She reaffirmed: “I can’t say that I think it’s ever influenced my clinical decision making.” By contrast, she went on to offer examples of cases where race and ethnicity might have an impact on patient care in her specialization. “People from Ireland have a much higher risk of celiac disease” and “In certain areas of the Middle East. . .consanguinity is more common, *so that comes up a lot*” (emphasis added). For these respective cases, she clarified that “I would never not screen someone because they’re not Irish,” and that “I do ask *everybody* that question: if there’s any chance that the parents of the child could be related by blood” (original emphasis). At one point, Dr. Freund offered an answer unrelated to my question and with a tone of realization which indicated a sudden (revised) recollection:

Actually! I do ask that [details about race and ethnicity] of my primary immunodeficiency patients. . .because sometimes there are diseases that are more common in certain sub-populations because of the Founder Effect where it was a small group of people who settled in a specific area. That’s mainly because for some of those diseases, you would be ordering a specific genetic test and clinically there could be a lot of different things and your initial screen could be changed by the ethnicity of the person.

Similarly, Dr. Edwards (Family Medicine) first explained that race and ethnicity do not “affect my clinical judgment . . .it’s just interesting. . .it’s more just things that you happen to note. . .I don’t think I’ve yet asked a patient ‘what’s your racial background’”. But Dr. Edwards’ views toward race became more explicit as we proceeded with the interview. It is not that Dr. Edwards abstains from collecting this information because it is irrelevant, but rather that she collects it *without* asking patients to self-identify “. . .largely because I can see with my eyes. . .their physical features. . ..” In other words, Dr. Edwards makes assumptions about her patients and actively uses this to inform patient care. In the example that Dr. Edwards offered, the patient “just looked” Chinese or Korean, so was suspected of being at a lower risk of diabetes.

Dr. Flores (Pediatrics) commented on how nurses and in-take administrators treat patients in the Intensive Care Unit (ICU) based on visual markers and other factors:

In the ICU, depending on your area code and where you came from, the nurses would give preferential care to the richer, more whiter [sic] families than the lower socio-economic ones. . .they would assume that CAS [Children’s Aid Society] needed to be involved with some families based on the way they dressed or what their teeth looked like even though they were wonderful parents, which I didn’t agree with. Some immigrants were assumed to be negligent or stupid when they both had PhDs. . .they just didn’t speak English. Things like that where people make cultural assumptions based on what they understand and they refuse to learn how other people do it.

The “preferential treatment” that he described is not far removed from the racist undertones of Dr. Williams’ (MedScope Editor) patient observation in the opening quote of this article regarding the association between a Latina schoolgirl and oral sex.

The visual markers also account for Dr. Roberts’ (Pediatrics) approach that was explained with the example of a patient with African ancestry: “If I think the family is from an African country, I can usually see that by the color of their skin”. I asked Dr. Roberts if his answer implied that Africans have a specific skin color. “Yes, usually black, but it doesn’t always need to be asked,” he responded. “[S]ometimes it’s just obvious, like if I’m thinking that a certain disease or condition is more prevalent in an African Canadian population, then I can tell if the person is Black or not.” The implication, therefore, is not only that race can be assessed by sight, but that “African equals Black.”

### I am an African

I am an African who does not appear Black. I positioned myself in this study because my personal experiences with race underscore its social construction and provided helpful lines of questioning for the interviews. I am an African by virtue of being born on the continent. But as someone born into apartheid South Africa, I was classified as “Indian,” only to voluntarily (but illustratively) amend this to “White” in 2013. Because this illustrates the social construction of race, practitioners were each asked to engage in a role play with me as the patient and them as the consulting doctors. The premise for the role play was: *If I were your patient and you needed to know my race, how would you go about collecting this information from me?*

The aim of the role play was to understand how the healthcare practitioners, without a PoC tool handy, would uncover details about my race (if applicable) for the purposes of diagnosis and/or treatment. Practitioners’ opening questions varied from “What part of the world are your parents or your parents’ parents from?” and “Where were you born?” to “What is your ancestry?” and “What is your last name?” Under different circumstances, the point of entry into uncovering my race might not matter, but in my case, the trajectory of each question led to a different conclusion: Indian, South African and White.

Dr. Flores (Pediatrics) and Dr. Patel (Dermatology) respectively relied on inferences from my surname and visual characteristics to conclude that “That’s an Indian surname” and “You look like me, so you’re clearly Indian!.” Not only does this approach overlook that my great-grandfather *changed* his surname (which I inherited) to win political favor with the British colonizers, but also that the way I look to others is not necessarily consistent with my self-identification. It was only Dr. Jordan (Family Medicine), whose starting point was my physical location at the time (Canada), who was able to eventually parse differences between skin pigment (brown) and self-identification (White). The question, “Would you mind telling me if you were born in Canada?,” was used as an invitation for a discussion about my background more broadly: how long I had been in Canada, where my parents were born, how I self-identified etc. She explained that “. . .it would be important for us to consider your skin color as well as how you self-identify because in your case those two things are different.”

### Interpreting “race” and “ethnicity” in MedScope content

If interpretations of race and ethnicity can impact an understanding of a patient, and potentially their diagnosis and treatment, then this brings serious attention to how healthcare practitioners engage with this information in PoC tools. When presented with different examples of arguably prejudicial framings of MedScope content, Dr. Williams (MedScope Editor) thought aloud in reflection of MedScope’s uses of race and ethnicity:

There is absolutely the potential for those kinds of biased interactions to go on between the patient and the physician and, yes, if we link a specific disease to a racial or ethnic group are we thereby stereotyping and are we potentially causing (a) the physician to make incorrect assumptions about their patient, (b) the physician to ask questions that might damage their relationship.

I took this matter to practitioners in an effort to gauge the connections between their own practices and MedScope content. In one example, Dr. Hince (Pediatrics) convincingly stated in the role play above that, “you would not be at high risk” of having a child with significant bilirubin [jaundice] levels by virtue of being of South African descent. This is consistent with MedScope that lists this condition as being most severe in people of “East Asian race.” When I asked her why my country of birth would remove my risk, Dr. Hince responded thoughtfully after a long pause, “umm. . .that’s a good question because I’m not sure if people of South African descent are also at high risk.” Dr. Hince’s initial response was to conform to PoC guidelines that she had memorized, but her self-doubt only emerged as a consequence of my follow up.

MedScope states that “Native Americans/Inuits have higher rates of AOM [Acute otitis media] *than* persons of white or black race” (emphasis added). I asked Dr. Zhao (Family Medicine), who used to work with Inuit populations in northern Canada, if the comparison in this insight—Native Americans/Inuit *versus* white or black race—is clinically helpful. Drawing attention to Population A *in comparison to* Population B arguably offers little indication about how susceptible Population A might be to the condition. “Yes, you’re right. . .the wording should be different,” he responded. “There should be a lot more precision and care about how these things are worded. . .I will be straight with you, PoC tools are no different to how I was trained in medical school [in the 1980s]. I was taught *totally* to racially profile”. . .I would say that the [MedScope] wording around that is very 1950s” (original emphasis). Dr. Zhao went on to argue that, “All [MedScope] is doing is just importing the racial profiling that I was taught in 1983.”

If Dr. Zhao was critical of racial profiling in PoC tools, then Dr. Roberts (Pediatrics) was comparatively in favor of this practice. Dr. Roberts explained that one preventative measure for patients at high risk of Type 2 Diabetes is leading healthy lifestyles. Noting that MedScope presents “African American and Hispanic patients” as having a higher prevalence of this condition, I questioned if it would be more appropriate to remind *all* patients to lead healthy lifestyles rather than directing this message specifically to these populations. While Dr. Roberts agreed with this, she noted the value of profiling patients by linking the potential negative outcomes of Type 2 Diabetes to race or ethnicity: “. . .sometimes it has more weight to it if you frame it in terms of preventing something that is more tangible than just preventing a bad outcome afterward”. Similar to these racial “scare tactics,” Dr. Edwards (Family Medicine) used diabetes as an example in her interview to make a point about the value of race and ethnicity. Although she believed that information about race and ethnicity in PoC tools is only incidental, she backtracked to state, “the one thing where [race and ethnicity] are important are [sic] for screening. For example, if an individual of one ethnic group is at higher risk of diabetes, we might start screening for diabetes at a younger age. But for someone of a different ethnicity, we might start screening them at a later age because it’s just not necessary to start younger”.

While Dr. Edwards routinely acts on information about race and ethnicity in PoC tools, Dr. Bugwandeen (Emergency Medicine) errs on the side of caution. He argued that relationships in PoC tools between medical conditions and race and ethnicity should *only* be considered in cases where “conditions that are very specific to. . .[certain] ethnicities” are known, such as with Tay Sachs Disease because the risk of failing to act is greater than pre-emptive treatment. MedScope includes the likely risk factors for Tay Sachs Disease as “Ashkenazi Jewish descent. . .[and] French Canadians living in Eastern Quebec (eastern St. Lawrence River Valley) or New England.” Dr. Bugwandeen conceded that patients of these populations who present “non-specific symptoms” should be treated based on the MedScope risk factors that are shared with other leading PoC tools. Non-specific symptoms are symptoms that a practitioner cannot directly observe or that are not reasonably linked to known health problems. In these cases, he argued, “the risk of giving an antibiotic is not that great compared to the risk of missing [the condition] such as Sickle Cell Anemia”.

Similarly, Dr. Flores (Pediatrics) only supported readings of race and ethnicity in PoC tools in exceptional cases provided that observed trends of race and ethnicity are used “. . .not to segregate people but to help them.” Like Drs. Edwards (Family Medicine) and Bugwandeen (Emergency Medicine), Dr. Flores’ (Pediatrics) approach to risk (with the example of malaria) is that “[i]f I’m not sure I just give them the vaccine. The harm versus the benefit. . .the benefit is way up here and there’s very little harm.” But Dr. Flores was possibly the most cautious of all the practitioners on the topic of race and ethnicity; he kept emphasizing the need to selectively apply this information in patient care. One of his main reasons for this is the algorithmic construction of PoC tools: “the algorithms. . .the guidelines. . .[construct] the optimum person and what you’re supposed do, but very few patients are the optimum person.” On this topic, Dr. Flores was most concerned about the pairing of Nurse Practitioners (NPs) with algorithms because many NPs rely on algorithmically sorted diagnoses and treatment plans. While doctors have the training and experience to question PoC data, NPs, he argued, more frequently accept the algorithmic suggestions at face value.

## Discussion

### Operationalizing race and ethnicity

The inconsistency across healthcare professionals’ conceptual understandings of “race” and “ethnicity” was unexpected. Because these terms are widely used in medical literature and public health, and are even searchable in PoC tools, it would be reasonable to expect a level of consistency in definition and interpretation. By contrast, in this study interpretations of race ranged from ancestry and genetics to location and skin color, while interpretations of ethnicity ranged from genetics and class to country of origin and being used interchangeably with race. To be clear, PoC tools cannot easily offer definitions to frame race and ethnicity because of how inconsistent the usage is in the medical literature from which algorithms extract findings to generate PoC insights. To complicate this matter, the lack of consistency in how practitioners understand race and ethnicity reinforces the socially constructed nature of the terms despite their use in medical literature as medically objective fact. This therefore adds to the argument that algorithms, by design, are not the only factor in shaping the uses and constructions of race and ethnicity in healthcare. What is clear, however, and consistent with the PHCRP, is that when algorithmic biases are combined with practitioners’ subjectivities—sometimes prejudicial—systemic differences in applying race and ethnicity are reproduced. While some practitioners were content with relying on their perceptions of the physical presentation of a patient’s race and ethnicity, others acknowledged the shortcomings of these concepts both in exercises of operationalization and application.

The conflation and/or inconsistent uses of race and ethnicity in patient care and medical literature is not merely an issue of semantics. For example, the inconsistent uses of race and ethnicity in medical literature could affect which research is captured in automated data collection for presentation in PoC tools. If an automated search tool is designed to scope the literature for “race” but some studies use “ethnicity,” then those findings might not be reported in the tool, thereby impacting the information presented in the app. Similarly, “race” and “ethnicity” have different social meanings, so a patient might not identify their race consistently with their ethnicity. The information that patients provide to healthcare practitioners could therefore misinform which medical conditions are associated with each patient.

When a practitioner assumes a patient’s race or ethnicity without the patient’s input and then aligns it with an algorithmically derived set of risks from a PoC tool, the practitioner’s assessment is likely to deepen racial stereotypes about social behavior and cultural practice.

### Healthcare practitioners’ prejudices

If a patient’s skin color is assumed to indicate place of origin, then it is unclear how many generations of settlement entitle one to associate their origins with a given location. This was evident in my interviews with healthcare practitioners who had difficulty aligning my country of birth (South Africa) and assumed skin color (black) with my actual skin color (brown), official race (white) and ancestry (India). Most of my answers to questions about my race in the role play established me in the eyes of practitioners as South African, Indian, or White indicating further inconsistency in the face of PoC content that presents such categories objectively. Many practitioners had the expectation that a South African should be “Black” and that one’s surname or accent indicates ancestry. But the assumption that a name or how one looks or sounds indicates place of origin, behavior or habits demonstrates the application of racial stereotypes in healthcare with or without PoC tools.

Perhaps most astonishing was Dr. Williams’ (MedScope Editor) acknowledgment that she previously aligned a patient’s physical symptom (sore throat) and assumed race/ethnicity (Latino) with a social behavior (oral sex). Further, the same practitioner confessed that the company she works for is “sloppy” when it comes to race and ethnicity (Personal Interview with Dr. Williams, 2018). When I pointed out how this aligns with the racism in MedScope content, she responded hesitantly and reflectively, “Why are we [MedScope] lumping together a specific behavior. . .with a class of persons that are [sic] at high risk?”. Not able to justify this, she admitted “Point well taken” (Personal Interview with Dr. Williams, 2018).

The racism that Dr. Flores (Pediatrics) highlighted about patient in-take in the ICU provides a starting point for future research into how hospital technologies (beyond PoC tools) might reinforce broader trends of institutional racism. This will also provide a further site to test the PHCRP. In this direction of analysis, what this study demonstrates is that when practitioners’ prejudices are combined with the racism that is embedded in PoC tools it becomes evident that personal bias, “herd mentality” and technological determinism are interlinked in examples of institutional racism in S.E. Ontario healthcare.

## Conclusion

In this article, I provided an empirical understanding of how healthcare practitioners use information in PoC tools about race and ethnicity to shape patient care, and explored how this relates to practitioners’ pre-existing stereotypes about their patients’ attributes. A PHCRP framework was used to foreground the social and health issues that emerged in the analysis, particularly with the overall demonstration of how the biological racism that is embedded in PoC algorithms interacts with healthcare practitioners’ own biases toward race and ethnicity. This framework also helps to show how racial bias in healthcare further contributes to the historical mistreatment of racialized populations.

The key findings in the analysis are as follows: firstly, the contested meanings of race and ethnicity in medical literature and PoC tools are consistent with those of the practitioners who were interviewed. While many practitioners defended biological explanations of race and ethnicity, the inconsistency in responses was ironically reflective of the social and political contestations of the terms. These sites of contestation—medical literature, PoC tools and practitioner bias—cumulatively contribute to racial prejudices in healthcare. Secondly, healthcare practitioners use PoC tools to inform patient care and diagnosis. However, in view of the contested constructions of race and ethnicity in PoC tools, participants’ lack of meaningful justification for their PoC tools of choice is of concern. Most concerning are the suggestions of a “herd mentality,” and evidence that technological determinism drives the hopes of PoC tools in patient care. Thirdly, when practitioners’ pre-existing prejudices (such as sore throat + Latino girl  = oral sex) are viewed alongside pithy conclusions about race and ethnicity in PoC tools (such as Latinos and sexually transmitted diseases), prejudiced interpretations of racialized patients are deepened and more acute. Fourthly, some practitioners defended the use of race and ethnicity in medical practice on the grounds that these concepts make patients take their conditions more seriously. Relatedly, some believed that acting on PoC data—even if the presentations are racist—is good medical practice because the benefits of doing so outweigh the risks. If practitioners consider PoC tools to be reliable despite their embedded racism then this leads to broader questions for future research about how race and ethnicity are dealt with in medical training, curriculum design in medical school, and practical application. When practitioners accept PoC content at face value even when the recommendations about race and ethnicity might be questionable, then this can reify the racism in PoC content and negatively impact patient care. While biological views might be well-intentioned, they unintentionally contribute to a broader discourse of racial subordination and health inequity (Roberts, 2008: 538).

There is scope for many fields of enquiry to tackle the negative contributions of PoC tools and practitioner prejudice in contributing to health inequities. While Noble (2018: 4) notes that “[i]t is impossible to know when and what influences proprietary algorithmic design, other than that human beings are designing them and that they are not up for public discussion, except as we engage in critique and protest,” the situation seems somewhat different in the case of PoC tools. What *is* possible to know about the algorithms that power PoC tools is that medical research plays a key role since it is published findings in the literature that algorithms surveil daily. Therefore, to extend Noble’s point about critique, one can add that reenvisioning methodologies in medical research that take into account the subjectivities of race and ethnicity will alter the information that literature surveillance algorithms detect for PoC tools. Until then, it seems that the combination of automated literature surveillance with healthcare practitioners’ pre-existing prejudices will contribute to the deepening of racial disparities in healthcare.

## Notes

1. In this article, the preference is to capitalize the first letter of all race groups (e.g. “Black”). However, lowercase is used when the word is (i) quoted as such from another source, and (ii) in reference to a skin color as opposed to a racial category.

2. I am unable to provide direct URLs to this content as it is derived from an institutional subscription to the PoC tool.
